# Differential alternative splicing between hepatocellular carcinoma with normal and elevated serum alpha-fetoprotein

**DOI:** 10.1186/s12920-020-00836-4

**Published:** 2020-12-28

**Authors:** Young-Joo Jin, Habtamu Minassie Aycheh, Seonggyun Han, John Chamberlin, Jaehang Shin, Seyoun Byun, Younghee Lee

**Affiliations:** 1 Department of Biomedical Informatics, University of Utah School of Medicine, Salt Lake City, UT USA; 2 Division of Gastroenterology, Department of Internal Medicine, Inha University Hospital, Inha University School of Medicine, Incheon, South Korea; 3 Huntsman Cancer Institute, University of Utah School of Medicine, Salt Lake City, UT USA

**Keywords:** Hepatocellular carcinoma, Alpha-fetoprotein, Alternative splicing, RNA-sequencing

## Abstract

**Background:**

Serum alpha-fetoprotein (AFP) is the approved serum marker for hepatocellular carcinoma (HCC) screening. However, not all HCC patients show high (≥ 20 ng/mL) serum AFP, and the molecular mechanisms of HCCs with normal (< 20 ng/mL) serum AFP remain to be elucidated. Therefore, we aimed to identify biological features of HCCs with normal serum AFP by investigating differential alternative splicing (AS) between HCCs with normal and high serum AFP.

**Methods:**

We performed a genome-wide survey of AS events in 249 HCCs with normal (n = 131) and high (n = 118) serum AFP levels using RNA-sequencing data obtained from The Cancer Genome Atlas.

**Results:**

In group comparisons of RNA-seq profiles from HCCs with normal and high serum AFP levels, 161 differential AS events (125 genes; ΔPSI > 0.05, FDR < 0.05) were identified to be alternatively spliced between the two groups. Those genes were enriched in cell migration or proliferation terms such as “the cell migration and growth-cone collapse” and “regulation of insulin-like growth factor (IGF) transport and uptake by IGF binding proteins”. Most of all, two AS genes (*FN1* and *FAM20A*) directly interact with AFP; these relate to the regulation of IGF transport and post-translational protein phosphorylation. Interestingly, 42 genes and 27 genes were associated with gender and vascular invasion (VI), respectively, but only eighteen genes were significant in survival analysis. We especially highlight that *FN1* exhibited increased differential expression of AS events (ΔPSI > 0.05), in which exons 25 and 33 were more frequently skipped in HCCs with normal (low) serum AFP compared to those with high serum AFP. Moreover, these events were gender and VI dependent.

**Conclusion:**

We found that AS may influence the regulation of transcriptional differences inherent in the occurrence of HCC maintaining normal rather than elevated serum AFP levels.

## Background

Hepatocellular carcinoma (HCC) is the sixth most common cancer in the world and the fourth leading cause of cancer-related deaths [1]. The prognosis of HCC is mainly determined by the tumor stage at time of discovery. However, most patients do not complain of symptoms before the HCC has progressed considerably [2], and this makes early diagnosis of HCC difficult. Moreover, patients diagnosed at an advanced stage usually miss the optimal window for receiving curative treatment, and their prognosis is ultimately very poor [2, 3]. Thus, in order to improve the prognosis of HCC patients, it is important to detect HCC early and to apply curative treatment in the early stages.

It was reported that early diagnosis using liver ultrasonography (USG) and serum alpha-fetoprotein (AFP) reduced the mortality rate of HCC patients by 37% [4]. Based on this result, liver USG at 6-month intervals is generally recommended for surveillance in high-risk groups (liver cirrhosis or hepatitis B or C), either alone or in combination with serum AFP testing [5–9]. For serum AFP, the standard cut-off value for maintaining appropriate sensitivity and specificity in HCC surveillance was initially reported to be 20 ng/mL [10]. However, some HCC patients show serum AFP levels below 20 ng/mL [11] and thus can escape early diagnosis despite receiving HCC surveillance tests using USG and serum AFP. Recently, there has been doubt about the usefulness of AFP testing for early diagnosis of HCC [5–7] but its utility remains in areas where the prevalence of HCC is high, as reflected in some guidelines for HCC surveillance [8, 9]. Although several studies have been performed to evaluate the clinical features of patients or tumor characteristics in the cases of HCC with normal (< 20 ng/mL) serum AFP levels [12–14], the molecular mechanisms underlying this feature remain to be determined. Given that newer biomarkers have yet to be developed that are more satisfying in HCC surveillance, it is necessary to understand the molecular biology of these HCCs with normal serum AFP levels.

In this study, we performed a genome-wide analysis to identify differential alternative splicing (AS) events between HCCs with normal (< 20 ng/mL) and high (≥ 20 ng/mL) serum AFP levels using RNA-sequencing data for tumor tissues obtained from The Cancer Genome Atlas (TCGA) database. Furthermore, we investigated the potential functions of genes with identified AS events and their association with HCC development (Additional file 1: Figures S1−S5).

## Methods

### Study population

**Fig. 1:**
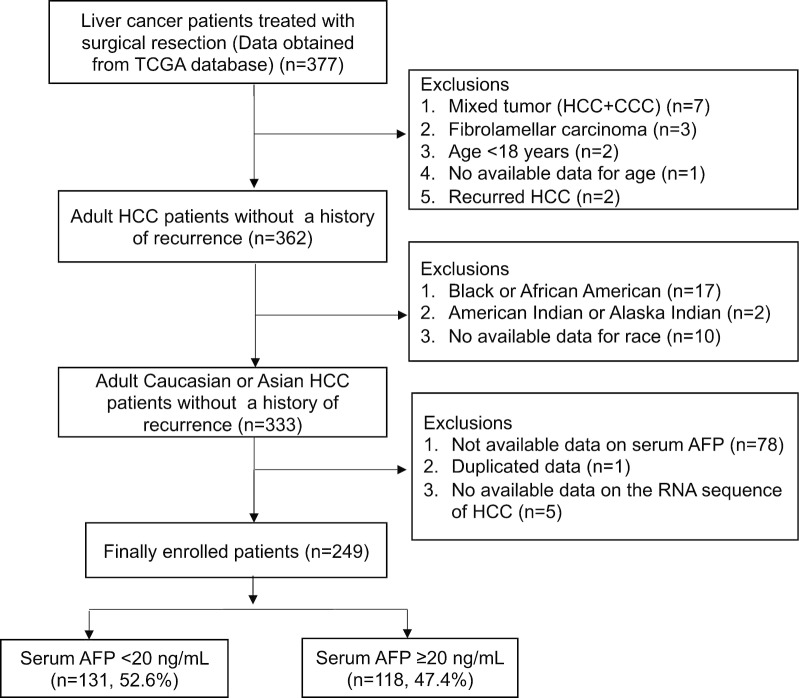
Flowchart of patient enrollment and exclusions. Of the initial 377 patients, 249 were enrolled in this study

A total of 377 liver cancer patients were retrospectively analyzed, and their clinical data and tumor RNA sequencing data were obtained from TCGA (Fig. 1). The patients underwent surgical resection (n = 376) or liver transplantation (n = 1) for treatment of liver cancer between 1995 and 2013. Of these 377 patients, 15 were excluded due to having mixed types of hepatocholangiocarcinoma (n = 7), fibrolamellar carcinoma (n = 3), age of less than 18 years (n = 2), no available age data (n = 1), or recurred HCC (n = 2). Of the remaining 362 patients with primary HCC, 29 were also excluded due to the small number of the patients being Black or African American (n = 17), being American Indian or Alaska Indian (n = 2) or having no available data for race (n = 10). In addition, 84 patients were excluded due to having no available data for serum AFP (n = 78), RNA sequencing (n = 5), or duplication data entry (n = 1). Thus, 249 primary HCC patients with normal serum AFP (< 20 ng/mL, n = 131, normal/low AFP group) and elevated serum AFP (≥ 20 ng/mL, n = 118, high AFP group) were finally enrolled in this retrospective study (Additional file 2: Table S1). HCC was pathologically diagnosed in all patients.

### Clinical data

Clinical data for study subjects were obtained from TCGA; the collected data included age, gender, race, causes of HCC, presence of liver cirrhosis (LC), serum AFP, albumin, prothrombin time (PT), bilirubin, Child-Turcotte-Pugh (CTP) classification, and tumor stage (American Joint Committee on Cancer, AJCC). LC was pathologically diagnosed based on the Ishak fibrosis scoring system using surgically obtained peritumoral normal liver tissue [15, 16]. Because tumor tissues were recruited over a period of time, tumor stages as initially recorded were based on different versions of the AJCC staging system, ranging from the 4th to 7th; thus, in this study, all stages were adjusted to the AJCC 7th version.

### Identification of alternative splicing events

RNA sequencing data, which was available for all enrolled patients (n = 249), was assessed to identify AS patterns in tumor tissues. We downloaded raw paired-end reads of RNA-seq Level 1 data (FASTQ files of study subjects) from the online Genomic Data Commons (GDC) data portal (http://gdac.broadinstitute.org/) in October 2018. We mapped the reads to the reference genome (i.e. release 75, GRCH 37.75 based on the hg19 reference sequence) using STAR v2.5 [17]. The proportions of uniquely mapped reads were greater than 80%. We detected AS events and quantified their expression as *percent spliced in* (PSI, ΔPSI or Δψ) using rMATs v3.2.5 [18]. PSI is a ratio of inclusion reads that indicates how efficiently sequences of interest are spliced into transcripts [19]. Analytically PSI can be represented as:

$$PSI = \frac{IR}{{IR + ER}}$$

where IR is the number of inclusion reads and ER that of exclusion reads. ΔPSI is then the difference between the PSI values of the two groups under study. When identifying AS events differentially expressed between groups, ΔPSI > 5% with FDR < 0.05 was considered statistically significant [18]. All AS events were assessed: exon skipping (ES), intron retention (IR), alternative 3′ splicing site (A3SS), alternative 5′ splicing site (A5SS), and mutually exclusive exons (MXE). Gene-level expression in transcripts per million (TPM) was calculated by RSEM version 1.3.0 [20], and identification of differential gene expression was performed with the *limma* R package, version 3.38.3 [21], using as covariates AFP status, gender, and the presence or absence of vascular invasion.

### Protein–protein interaction network analysis

Protein–protein interaction networks (PPINs) were created using genes from the STRING *v*.11.0 dataset (http://string-db.org/) [22]. In the absence of disconnected nodes, only the first gene interaction with a relaxed highest confidence > 0.7 was selected. PPINs were visualized using Cytoscape v3.7.2 [23].

### Functional gene set enrichment analysis

Gene set enrichment analysis of the identified AS genes in terms of canonical pathways was conducted with the ConsensusPathDB-human database (CPDB) [24]. FDR < 0.05 was considered statistically significant.

### Clinical association test

Within identified AS events, we investigated whether AFP-dependent splicing events had any relevant clinical features. First, we divided the 249 cases into two groups according to PSI values (i.e. low and high PSI) of AS exons through K-means clustering. With these two groups, we performed a Kaplan–Meier survival analysis to evaluate overall survival (OS) outcome according to PSI value. Other important clinical features of gender and vascular invasion were evaluated using Wilcoxon t-tests.

### Statistical analysis

Patient baseline characteristics were described as mean ± standard deviation or as frequency. The significance of differences between categorical or continuous variables was determined by *Chi*-square test, Fisher’s exact test, or Student’s *t* test, as appropriate. Two-tailed *p* values of < 0.05 were considered statistically significant. In addition, to avoid false positives during multiple testing for DGE, we used a corrected *q* value obtained after FDR correction. A corrected FDR of < 0.05 was considered to be statistically significant. Statistical analyses of clinical data were performed using SPSS v19.0 (SPSS Inc, Chicago, IL, USA) [25]. All other analyses were conducted with R version 3.5.1 [26].

## Results

### Baseline characteristics of patients

The clinical characteristics of study subjects are shown in Table 1. The mean ages of normal and high AFP groups were 60 (standard deviation (SD) ± 13 years) and 59 years (SD ± 13 years), respectively (*p* = 0.317). The proportion of male patients was significantly higher in the normal AFP group (*p* = 0.005). Between the two AFP groups, there was no statistically significant difference in white/Asian race ratio, etiology, presence of LC, and AJCC 7th TNM stage (*p* values for all > 0.05). In the normal AFP group, vascular invasion was less likely than in the high AFP group (*p* = 0.008).

**Table 1:** Baseline clinical characteristics of study subjects (n = 249)

Variables (n = 249)	AFP < 20 ng/mL (n = 131, 52.6%)	AFP ≥ 20 ng/mL (n = 118, 47.4%)	*p* value
Age (year)^§^	60 ± 13	59 ± 13	0.300
Gender (male), n (%)	96 (73.3)	66 (55.9)	0.005
Race, n (%)			0.376
White/Asian	72/59 (55.0/45.0)	58/60 (49.2/50.8)	
Cause of HCC, n (%)^a^	(n = 129)	(n = 117)	0.384^+^
HBV	41 (31.8)	47 (40.2)	
HCV	20 (15.5)	11 (9.4)	
Alcohol	26 (20.1)	18 (15.4)	
Mixed (HBV + HCV)	2 (1.6)	3 (2.6)	
Others	40 (31.0)	38 (32.5)	
Liver cirrhosis, n (%)^b^	(n = 96)	(n = 70)	0.744
Presence	36 (37.5)	24 (34.3)	
Vascular invasion, n (%)^c^	(n = 121)	(n = 110)	0.008^+^
Absence/presence	89/32 (73.6/26.4)	62/48 (56.4/43.6)	
AJCC,7th, pTNM stage, n (%)^d^	(n = 126)	(n = 108)	0.468^+^
I	76 (60.3)	57 (52.8)	
II	30 (23.8)	26 (24.1)	
III (A/B/C)	18 (14.3)	21 (19.4)	
IV (A/B)	2 (1.6)	4 (3.7)	

Liver cirrhosis was pathologically defined based on Ishak fibrosis score system

AFP, Alpha-fetoprotein; HBV, hepatitis B virus; HCV, hepatitis C virus; LC, liver cirrhosis; AJCC, American Joint Committee on Cancer

^*^P values were calculated using the *t*-test, the *Chi*-square test, or Fisher’s exact test, as appropriate

Data for a, b, c, and d were available in 246, 166, 231, and 234 patients, respectively

^§^Mean ± standard deviation

^+^Fisher’s exact test

### Comparability of AFP serum protein level and gene expression

**Fig. 2:**
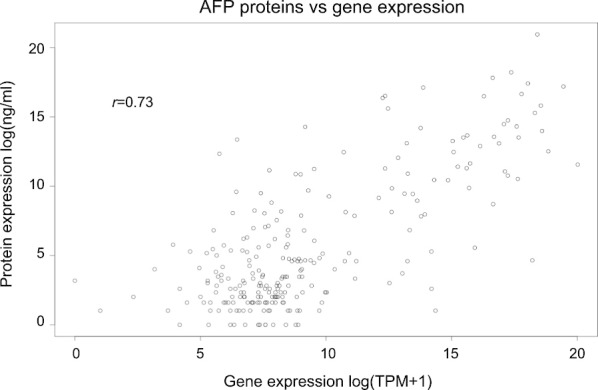
AFP protein level is primarily explained by variation in *AFP* gene expression. Samples were grouped according to AFP status and fold changes calculated between “high AFP” and “normal AFP” samples. TPM, transcripts per million

**Fig. 3:**
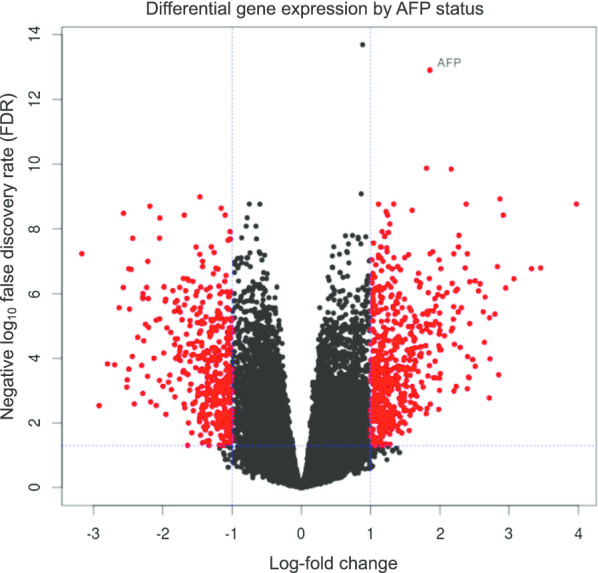
Differential gene expression. Differential gene expression analysis was performed with the *limma* R package, version 3.38.3. Samples were grouped according to AFP status, and fold changes calculated between “high AFP” and “normal AFP” samples. Absolute fold changes of 2 with FDR = 0.05 are marked as blue lines. Significant genes are highlighted in red. The *AFP* gene is labeled. As expected, it shows the largest fold change of 3.97 (adjusted *p* = 1.72 × 10^–9^). Differential expression according to AFP status and additional covariates ‘vascular invasion’ and ‘gender’ are summarized in Additional file 3: Table S2

In order to investigate whether *AFP* gene expression is comparable to serum levels of the corresponding protein product, *AFP* (ENSG00000081051) expression (TPM computed by RSEM) was compared to the serum AFP protein level (ng/mL, obtained from TCGA). To avoid bias due to high right-skew, the correlation coefficient was computed for log-transformed units. As shown in Fig. 2, as expected, *AFP* gene expression was positively correlated with AFP serum protein level (r = 0.73, *p* ≤ 2 × 10^–16^). Most of all, in high AFP patients, *AFP* is the most differentially expressed gene by absolute fold change and the six most significant (log value of fold change = 3.97, *p* = 1.72 × 10^–9^) when compared to low AFP patients (Fig. 3). Differential gene expression by AFP serum status is summarized in Additional file 3: Table S2.

### AS events differentially expressed between normal and high AFP groups

Having identified AFP-dependent gene expression (Fig. 2), we further investigated whether differences at the AS level were significant according to AFP level. We identified differential AS events between low and high AS groups using rMATs; these are summarized in Table 2, showing 161 differential AS events comprising 125 unique corresponding genes (ΔPSI > 0.05, FDR < 0.05). A more stringent cutoff of ΔPSI > 0.1 would have yielded only 16 AS events affecting 16 genes; we opted for the lower cutoff value in order to investigate more marginal differences between the two groups. This more detailed marginal signal comparison helps to investigate functional relationships between the groups. The distributions of each AS event type (ES, IR, A3SS, A5SS, and MXE) are summarized in Table 2 and detailed in Additional file 4: Table S3. As expected, ES and RI events were the most widely observed, followed by A5SS, A3SS, and MXE.

**Table 2:** Counts of AS events differentially expressed between the normal and high AFP groups

Alternative splicing types	Number of events	Number of corresponding genes
Exon skipping (ES)	104	79
Retained intron (RI)	27	26
Alternative 5′ splice sites (A5ss)	14	14
Alternative 3′ splice sites (A3ss)	13	13
Mutually exclusive exon (MXE)	3	3
Total	161	125

As stated above, we identified 125 genes with differential alternative splicing events in the context of AFP level. We then asked the question, “are these AFP level-dependent splicing events relevant to the mechanism underlying AFP levels in HCC patients?” We accordingly performed a multi-layered functional analysis at the network level combined with pathway analysis to interpret the relationship of molecular pathology to AFP level and performed a correlation test to evaluate the clinical implications of the identified AS events in HCC patients.

### Functional analysis of genes with differentially expressed AS events

**Fig. 4:**
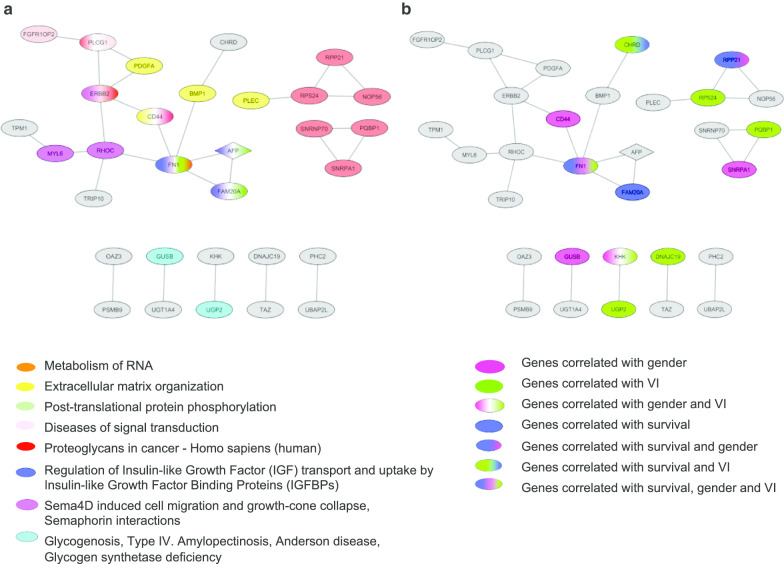
Functional analysis of identified AS genes. **a** Canonical pathways enriched in the PPIN of AS genes. **b** Clinical implications of AS genes

In an interaction network, proteins closer to each other are more likely to have related functions [27]. Proteins that are directly linked to a given protein (i.e. sharing a single network edge) are called "first interactors (first interacting groups)”. First interactors tend to be involved in the same disease or biological process [28]. We defined the first interactors of our 125 identified AS genes using the STRING database (relaxed highest confidence score ≥ 0.7) [29]. Of these, 31 genes were identified as having a known protein–protein interaction (PPI). The structure of this PPI network is illustrated in Fig. 4a. In order to investigate the functional association of the spliced genes with HCC molecular pathology, we further conducted functional enrichment analysis of the networked proteins over canonical pathways. Twenty genes from the PPI network (Additional file 1: Figure S1) were determined to belong to eight canonical pathways having significant enrichment, as illustrated in Fig. 4a. Most interestingly, protein products from two genes (*FN1* and *FAM20A*) directly interact with AFP, with these genes belonging to the pathway “regulation of IGF transport and uptake by IGFBPs” and to “post-translational protein phosphorylation” or “extracellular matrix organization.”

### Clinical association of differentially expressed AS events

The two AFP groups differed significantly in terms of gender and vascular invasion (VI) (Table 1), thus we tested the correlation of differentially expressed AS events with these variables. Among the 125 genes with differential AS events, 50 events (42 genes) and 34 events (27 genes) were gender and VI dependent, respectively as shown in Table 3 (more details in Additional file 5: Table S4). To further gain insight into the molecular connection between these factors and genetic pathways, we examined the gender- and VI-associated genes in the context of the entire 31genes PPIN, as shown in Fig. 4b. In the PPIN, six genes were associated with gender specifically; these consisted of three genes with ES events (*FN1, GUSB*, and *CD44*); two genes having RI events (*RPP21* and *SNRPA1*); and one having both ES and MXE events (*KHK*). Moreover, seven genes were associated with VI specifically; these consisted of four genes with ES events (*FN1, UGP2, DNAJC19*, and *RPS24*); one gene having A5SS events (*PQBP1*); one gene with both ES and MXE events (*KHK*); and one gene having both ES and A5SS events (*CHRD*). All told, two genes (*FN1* and *KHK*) were found to be associated with both gender and VI.

**Table 3:** Counts of differentially expressed AS events associated with gender and VI

Alternative splicing types	Gender		VI	
	Number of events	Number of corresponding genes	Number of events	Number of corresponding genes
Exon skipping (ES)	30	24	19	16
Retained intron (RI)	11	11	5	5
Alternative 5′ splice sites (A5ss)	5	5	5	5
Alternative 3′ splice sites (A3ss)	3	3	3	3
Mutually exclusive exon (MXE)	1	1	2	2
Total	50	42	34	27

We additionally evaluated the presence in the PPIN of genes having differential AS that were associated with patient survival. Of 125 genes, 18 showed association with survival (Additional file 6: Table S5); of those, four genes (*FN1, FAM20A, CHRD,* and *RPP21*) were present in PPI networks. Of these 4 genes, we conducted survival analysis more in detail for *FN1* and *FAM20A,* which were identified to directly interact with AFP in the present study (Fig. 4a). Notably, the estimated cumulative overall survival (OS) rate of patients with low levels of ES in *FN1* and FAM20A were significantly higher than those of patients with high PSI for *FN1* (*p* = 0.047) (Additional file 1: Figure S2A) and *FAM20A* (*p* = 0.024) (Additional file 1: Figure S2B), respectively.

For the two genes, we further analyzed the survival of patients according to higher PSI levels and gene-level expression. We grouped the 249 patients into high groups with PSI > 0.1 and 0.2 and low groups with PSI < 0.1 and 0.2 for each of two exons of *FN1* and one exon of *FAM20A*, then tested for differences in survival. However, there were no survival differences in the context of PSI level (*p* values for all > 0.05). We also explored the survival of patients according to gene-level expression. The estimated cumulative overall survival (OS) rate of patients with low expression of *FN1* was significantly higher than those of patients with high expression (*p* = 0.009) (Additional file 1: Figure S3A). However, there was no significant survival difference between patients with high and low expression of *FAM20A* (*p* = 0.489) (Additional file 1: Figure S3B).

## Discussion

In this genome-wide analysis, we identified 125 genes with AS events that were differently expressed in the context of HCC with normal (low) serum AFP when compared to HCC with high serum AFP. Two of these AS genes, *FN1* and *FAM20A*, had proteins that were identified to directly interact with AFP. Moreover, we found that in HCCs with normal serum AFP, *FN1* exhibited more differential expression of AS events (ΔPSI > 0.05), with exons 25 and 33 of *FN1* being more frequently skipped. Although the splicing level change is subtle, we believe that the observed splicing level change can be true as we found that gene-level expression did not differ based on AFP level. The present study is unique because we firstly imposed the possibility that AS may influence the regulation of transcriptional differences inherent in the occurrence of HCC with normal serum AFP level. These findings suggest that genome-wide analysis to identify differential AS events may be helpful for early diagnosis of HCCs showing normal (< 20 ng/mL) serum AFP. As *FN1* is also known to be overexpressed in HCC [30], we further evaluated the differential expression of *FNI* transcript isoforms between low and high AFP groups. As shown in Additional file 1: Figure S4, there were nine transcript isoforms annotated as having a protein product (Ensembl version GRCh37 release 100) and expressed in our dataset, of which one transcript (ENST00000357867) showed slightly different fold change (1.48, *p* = 0.011). We also verified the reliability of the AS quantification using another popular tools, which is MISO [31]. We measured the PSI by MISO and the measured PSI value was compared to the PSI from rMAT. We found that the PSI values from these two different tools were highly correlated (median correlation *r* = 0.70), verifying the reliability of the AS quantification between different methods. Especially, PSI value of 33rd exon and 25th exon of *FNI* from two tools were also highly correlated (*r* = 0.90 and *r* = 0.93, respectively), Additional file 1: Figure S5.

Together with abdominal USG, AFP has been used as a screening method in populations at high risk for HCC development [5–9] as well as for non-biopsy HCC diagnosis [5, 32] and the assessment of treatment responses [33]. However, not all HCC tumors show high serum AFP levels [11, 34], and with a cut-off of 20 ng/mL, the sensitivity of this method for the diagnosis of HCC is about 60% [10]. Thus, only liver USG is recommended as a screening method for early diagnosis of HCC in the USA, Europe, and Asia–Pacific countries where the prevalence of HCC is relatively low [5–7]. On the other hand, in Korea and Japan, where the prevalence of HCC is high, it has been recommended to perform liver USG and serum AFP measurement together for HCC surveillance [8, 9]. In order to not miss HCC patients with normal serum AFP, it is necessary to identify the features of these HCCs. Previous studies have reported associations of tumor size or the presence of PVT [35], but even HCCs with low serum AFP can exhibit large size, suggesting other factors must be associated with serum AFP level in HCC. Therefore, it is necessary to understand the differential molecular pathology between cases with normal or higher serum AFP levels.

Until now, no data has been reported that explains the underlying pathophysiologic mechanisms of serum AFP levels in HCC at the transcriptional level. In the present study, in order to interpret the molecular pathophysiology of HCC in relation to serum AFP level, we evaluated the association of gene expression with serum AFP protein. We found that *AFP* is the most differentially expressed gene when considering AFP status, and furthermore identified 161 AS events (125 genes) whose occurrence differed significantly between normal and high AFP groups. Interestingly, in the present study, two genes (*FN1* and *FAM20A*) were identified as encoding proteins that interact directly with AFP, and an ES event in *FN1* was found to be closely associated with the development of HCCs having normal serum AFP.

Moreover, the present study identified an association of high PSI for an ES event in *FN1* or *FMA20A* with poor prognosis of HCC patients, who underwent surgical resection. In light of these findings, *FN1* and *FAM20A* should receive further experimental investigation to evaluate whether either can be a potential candidate for diagnostic biomarkers for early diagnosis of HCC in high-risk population for HCC, but with normal serum AFP, and be prognostic predictors of post-surgical outcomes for HCC in them. The present study did not directly analyze the functional association of *FN1* and *FAM20A* with HCC prognosis, but our data may suggest AS to have an effect on the regulation of transcriptional differences with regard to the survival of HCC patients.

**Fig. 5:**
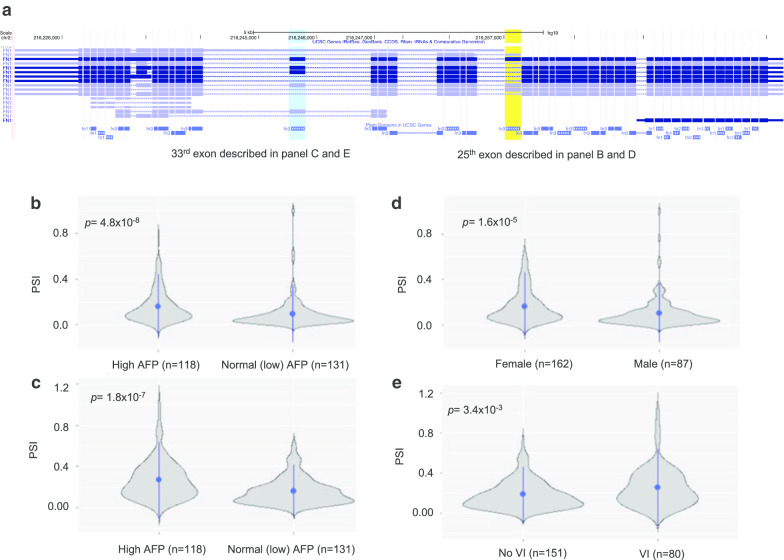
Case study: *FN1* gene. There are two exons more frequently skipped. **a** The gene overview, with skipped exons highlighted in yellow (25th exon, described in **b**, **d** and cyan (33rd, described in Panel C and E). The 25th (**b**) and 33rd exons (**c**) are more frequently skipped in low AFP HCC patients. Interestingly, the 25th exon tends to be more skipped in males compared to females (**d**), and the 33rd exon tends to be less skipped in VI (**e**)

The gene *FN1* encodes fibronectin (FN), a glycoprotein produced by hepatocytes and present in both plasma and cellular forms [36, 37]; its major functions are in cell adhesion, cell morphology, thrombosis, cell migration, and embryonic differentiation, as well as being part of the “extracellular matrix organization” pathway. FN is a modular protein composed of homologous repeats of three prototypical domains known as types I, II, and III [38]; of these, fibronectin type-III (FN3) is the largest and the most abundant in the protein [39–42].Previous studies have reported alternatively spliced mRNA variants of *FN1*, and these have been highly associated with HCC development [43, 44], but have not been investigated in the context of normal serum AFP level. In the present study, we found that two exons of *FN1* were more frequently skipped in HCCs with normal serum AFP than in those with high serum AFP. Figure 5a depicts the skipping of exon 25 and exon 33 (ENST00000354785 transcript isoform), highlighted in yellow and cyan respectively, showing significant mean PSI difference associated with serum AFP (Fig. 5b, c, respectively). These exons encode part of the FN3 domain region (PF00041) of the protein product. Moreover, *FN1* was also significantly associated with the clinical features of gender (25th exon only) and VI (33rd exon only) in HCC (Fig. 5d, e, respectively). While exon 25 was more skipped in male patients than female patients, exon 33 was more skipped in HCC without VI relative to cases with VI. These findings suggest that female patients with loss of FN3 domains due to skipping of exon 33 may be most likely to have HCCs with normal (low) serum AFP and without VI. Therefore, in these patients, HCC surveillance using liver USG with serum AFP testing may not be useful, and more careful inspection using liver CT or magnetic resonance imaging [45] may be needed for early detection of HCC.

In the present study, we investigated the relationship between AFP level and the functionality of the 125 genes having AS associated with HCC molecular pathology. Through functional enrichment analysis on the 31 genes having a known PPI with AFP, we identified eight enriched canonical pathways. Of particular note were *FN1* and *FAM20A*, identified to directly interact with AFP and to be part of the pathways “regulation of IGF transport and uptake by IGFBPs” and “post-translational protein phosphorylation” or “extracellular matrix organization.” It has been reported that IGF expression is associated with HCC development [46–48]. IGFBPs bind IGF ligands with high affinity and modulate the half-life and bioavailability of IGF-I/III [49]. Moreover, IGFBP activities are closely regulated by post-translational modifications and IGFBP proteases [50–54]. These findings suggest that further understanding AS of FN1 may provide insight into whether these AFP level-dependent splicing events are relevant to the development of HCCs with normal serum AFP. However, no direct clinical data has been reported associating *FAM20A* with hepatocarcinogenesis, and thus, the association of this gene with HCC development needs to be validated in a further study.

There are some limitations in this study. First, this study was retrospectively analyzed due to the nature of the data, and thus selection bias was inevitable. Second, validation using blood samples of HCC patients was not performed. However, we applied well-defined bioinformatics analysis methods to identify differentially expressed AS events between HCCs with normal and high serum AFP levels. Nonetheless, the outcomes of this study need to be validated using a well-designed prospective study in the future.

## Conclusion

In conclusion, in this genome-wide study, we found that AS of *FN1* was differently expressed in HCC with normal serum AFP compared to those with high serum AFP. Moreover, we found that skipping of exon 33 of *FN1* occurred more frequently in the normal AFP group than in the high AFP group, and less frequently in patients with VI. Interestingly, the PSI level of exon 33 is also associated with overall survival rate. Given that AS events associated with the development of HCC having normal AFP level has not been sufficiently investigated, we expect that our outcomes may provide helpful information for understanding the molecular characteristics of HCCs with normal serum AFP. In addition, if these results are validated in well-designed future studies, it may be possible to carry out individualized HCC surveillance in high-risk populations using a genome-wide study and consideration of serum AFP.

## Supplementary information

**Additional file 1: Figure S1.** Pathways significantly enriched in AS genes. **Figure S2.** Cumulative overall survival curve of patients according to PSI level in FN1 and FAM20A. Cumulative overall survival curve of patients according to PSI level of an exon skipping event in *FN1* (A) and an exon skipping event in *FAM20A* (B). These events are marked in orange color in the Additional file 6: Table S5. **Figure S3.** Cumulative overall survival curve of patients according to *FN1* and *FAM20A* gene expression. Cumulative overall survival curve of patients according to expression of an exon skipping event in *FN1* (A) and an exon skipping event in *FAM20A* (B). K-means clustering was used to divide the cases into two groups according to the gene-level expression value calculated by RSEM. With these two groups, we performed a Kaplan–Meier survival analysis to evaluate overall survival (OS) outcome according to gene expression. **Figure S4.** Expression of *FN1* mRNA transcripts between low and high AFP groups. We selected protein coding transcripts for which the median value of expression in total samples was more than zero, and compared expression between low (< 20 ng/mL) and high (> = 20 ng/mL) serum AFP groups with a *t-*test. Fold changes were calculated as the ratio of mean expression and tested by *t-*test. **Figure S5.** High correlation of PSI values between two different AS quantification tools. (A) A scatter plot of PSI level of 33rd exon in *FN1* calculated from x-axis (rMATs) and y-axis (MISO). The correlation is 0.900. (B) A scatter plot of PSI level of 25th exon in *FN1* calculated from x-axis (rMATs) and y-axis (MISO). The correlation is 0.928.

**Additional file 2: Table S1.** 249 TCGA patients enrolled in the study.

**Additional file 3: Table S2.** Differential gene expression between the normal and high AFP groups.

**Additional file 4: Table S3.** Significant AS events differentially expressed between the normal and high AFP groups.

**Additional file 5: Table S4.** Correlation of differentially expressed AS events with gender and VI.

**Additional file 6: Table S5.** Correlation of differentially expressed AS events with survival rate.

## Data Availability

All data generated or analyzed during this study are included in this published article (and its supplementary information files).

## Abbreviations

AFP: Alpha-fetoprotein
AJCC: American Joint Committee on Cancer
AS: Alternative splicing
CTP: Child-Turcotte-Pugh
DGE: Differential gene expression
GDC: Genomic Data Commons
HCC: Hepatocellular carcinoma
LC: Liver cirrhosis
OS: Overall survival
PT: Prothrombin time
PVT: Portal Vein Thrombosis
TCGA: The Cancer Genome Atlas
TNM: Tumor node metastasis
USG: Ultrasonography
VI: Vascular invasion
