# Supplementary material for: Differential alternative splicing between hepatocellular carcinoma with normal and elevated serum alpha-fetoprotein
Source: BMC Med Genomics. 2020 Dec 28;13(Suppl 11):194. doi: 10.1186/s12920-020-00836-4 (PMC7771076; doi:10.1186/s12920-020-00836-4)
Supplement: Supplementary file 1 — Additional file 1: Figure S1. Pathways significantly enriched in AS genes. Figure S2. Cumulative overall survival curve of patients according to PSI level in FN1 and FAM20A. Cumulative overall survival curve of patients according to PSI level of an exon skipping event in FN1 (A) and an exon skipping event in FAM20A (B). These events are marked in orange color in the Additional file 6: Table S5. Figure S3. Cumulative overall survival curve of patients according to FN1 and FAM20A gene expression. Cumulative overall survival curve of patients according to expression of an exon skipping event in FN1 (A) and an exon skipping event in FAM20A (B). K-means clustering was used to divide the cases into two groups according to the gene-level expression value calculated by RSEM. With these two groups, we performed a Kaplan–Meier survival analysis to evaluate overall survival (OS) outcome according to gene expression. Figure S4. Expression of FN1 mRNA transcripts between low and high AFP groups. We selected protein coding transcripts for which the median value of expression in total samples was more than zero, and compared expression between low (< 20 ng/mL) and high (> = 20 ng/mL) serum AFP groups with a t-test. Fold changes were calculated as the ratio of mean expression and tested by t-test. Figure S5. High correlation of PSI values between two different AS quantification tools. (A) A scatter plot of PSI level of 33rd exon in FN1 calculated from x-axis (rMATs) and y-axis (MISO). The correlation is 0.900. (B) A scatter plot of PSI level of 25th exon in FN1 calculated from x-axis (rMATs) and y-axis (MISO). The correlation is 0.928. [file 12920_2020_836_MOESM1_ESM.pdf]

Additional Files

Differential alternative splicing between hepatocellular carcinoma with normal and elevated serum alpha-fetoprotein

Young-Joo Jin[1,2,§], Habtamu Minassie Aycheh[1,§], Seonggyun Han[1,§], John Chamberlin[1], Jaehang Shin[1], Seyoun Byun[1], and Younghee Lee[1,3]*

The affiliations of authors:

[1]Department of Biomedical Informatics, University of Utah School of Medicine, Salt Lake City, UT, USA;
[2]Division of Gastroenterology, Department of Internal Medicine, Inha University Hospital, Inha University School of Medicine, Incheon, South Korea;
[3]Huntsman Cancer Institute, University of Utah School of Medicine, Salt Lake City, UT, USA

[§] Contributed equally

*Corresponding Authors: Younghee Lee, Ph.D.

Corresponding authors' address: Younghee Lee

Department of Biomedical Informatics

University of Utah School of Medicine

Salt Lake City, UT, USA

Corresponding authors' e-mail address: younghee.lee@utah.edu

Running title: Alternative splicing and AFP in hepatocellular carcinoma

Additional Files

Additional file 1 (.pdf):

Figure S1. Pathways significantly enriched in AS genes.

Figure S2. Cumulative overall survival curve of patients according to PSI level in FN1 and FAM20A. Cumulative overall survival curve of patients according to PSI level of an exon skipping event in *FN1* (**A**) and an exon skipping event in *FAM20A* (**B**). These events are marked in orange color in the **Table S4**.

Figure S3. Cumulative overall survival curve of patients according to *FN1* and *FAM20A* gene expression. Cumulative overall survival curve of patients according to expression of an exon skipping event in *FN1* (**A**) and an exon skipping event in *FAM20A* (**B**). K-means clustering was used to divide the cases into two groups according to the gene-level expression value calculated by RSEM. With these two groups, we performed a Kaplan-Meier survival analysis to evaluate overall survival (OS) outcome according to gene expression.

Figure S4. Expression of *FN1* mRNA transcripts between low and high AFP groups. We selected protein coding transcripts for which the median value of expression in total samples was more than zero and compared expression between low (<20 ng/mL) and high (>=20 ng/mL) serum AFP groups with a *t*-test. Fold changes were calculated as the ratio of mean expression and tested by *t*-test.

Figure S5. High correlation of PSI values between two different AS quantification tools. (A) A scatter plot of PSI level of 33rd exon in *FN1* calculated from x-axis (rMATs) and y-axis (MISO). The correlation is 0.900. (B) A scatter plot of PSI level of 25th exon in *FN1* calculated from x-axis (rMATs) and y-axis (MISO). The correlation is 0.928.

Additional file 2. (.xlsx): Table S1. 249 TCGA patients enrolled in the study.

Additional file 3. (.xlsx): Table S2. Differential gene expression between the normal and high AFP groups.

Additional file 4. (.xlsx): Table S3. Significant AS events differentially expressed between the normal and high AFP groups.

Additional file 5. (.xlsx): Table S4. Correlation of differentially expressed AS events with gender and VI.

Additional file 6. (.xlsx): Table S5. Correlation of differentially expressed AS events with survival rate.

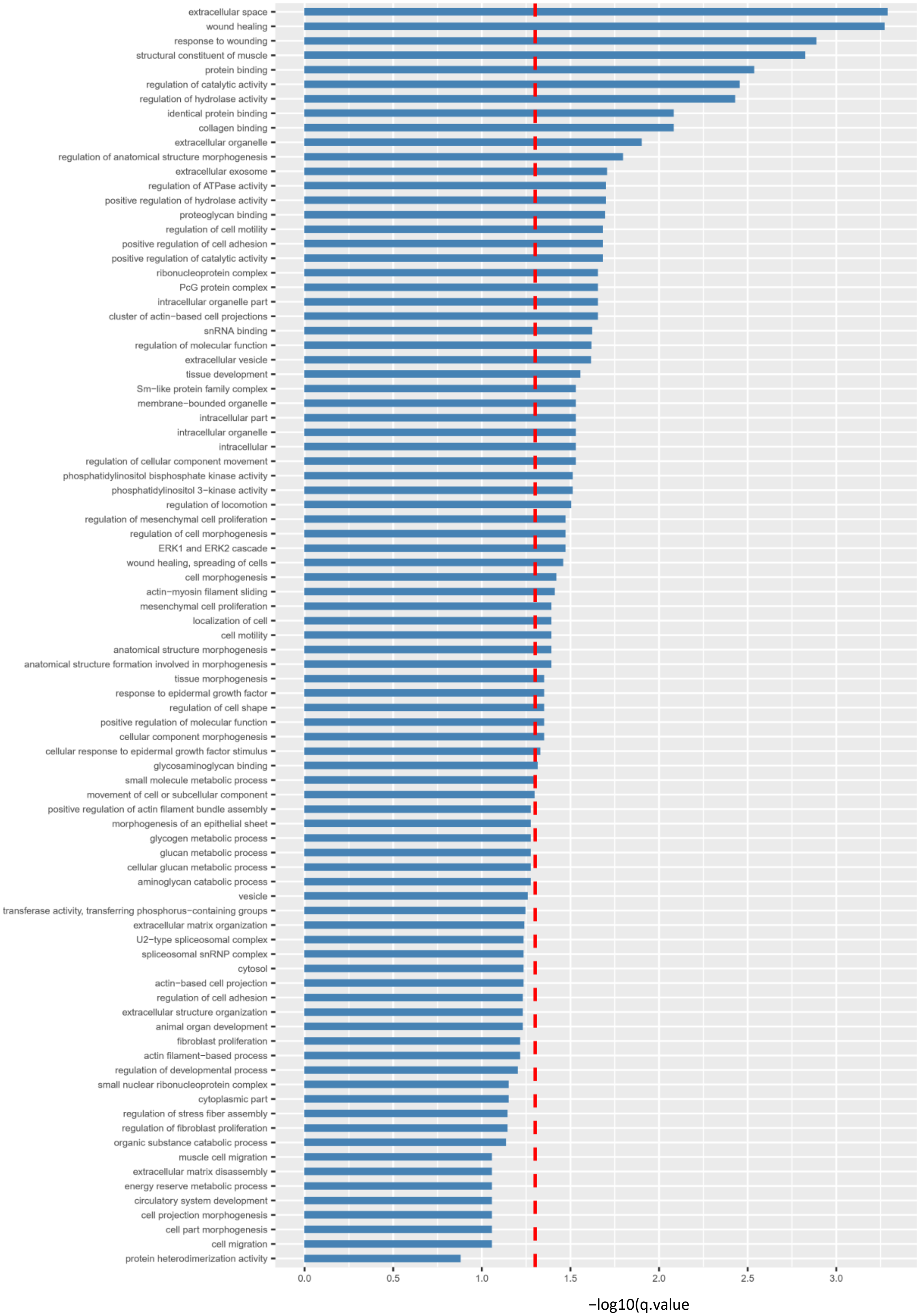

Figure S1. Pathways significantly enriched in AS genes

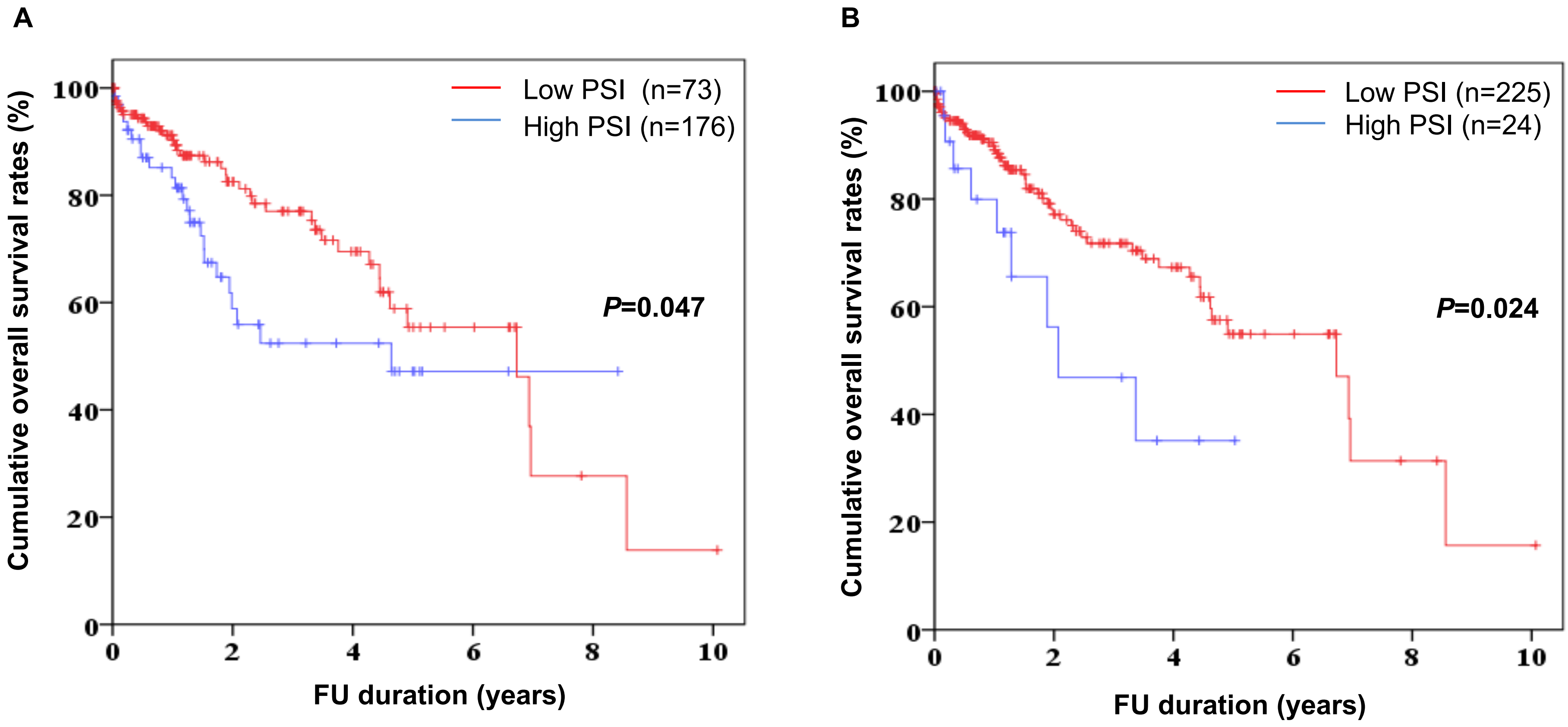

Figure S2. Cumulative overall survival curve of patients according to PSI level in FN1 and FAM20A. Cumulative overall survival curve of patients according to PSI level of an exon skipping event in *FN1* (**A**) and an exon skipping event in *FAM20A* (**B**). These events are marked in orange color in the **Table S5**.

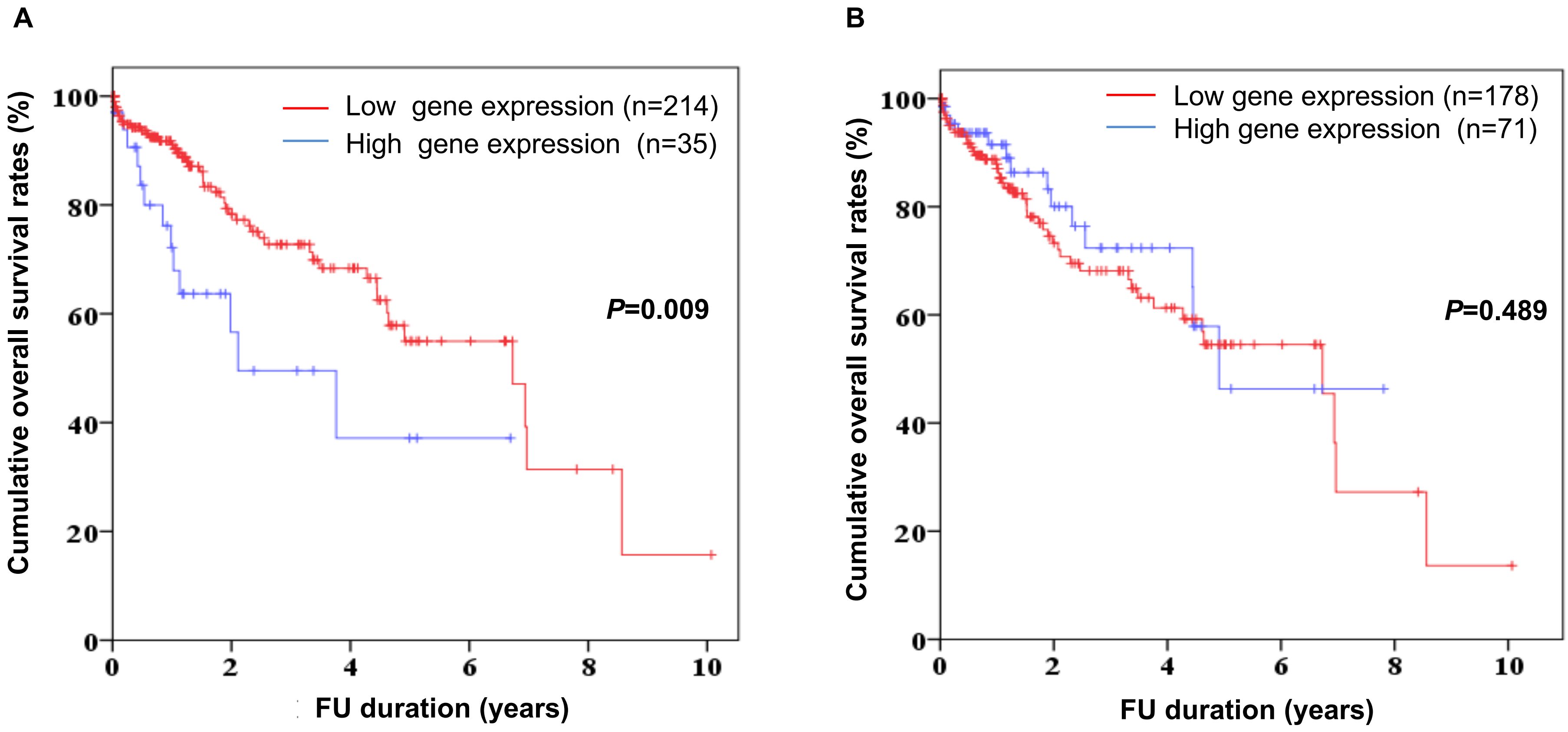

Figure S3. Cumulative overall survival curve of patients according to *FN1* and *FAM20A* gene expression. Cumulative overall survival curve of patients according to expression of an exon skipping event in *FN1* (**A**) and an exon skipping event in *FAM20A* (**B**). K-means clustering was used to divide the cases into two groups according to the gene-level expression value calculated by RSEM. With these two groups, we performed a Kaplan-Meier survival analysis to evaluate overall survival (OS) outcome according to gene expression.

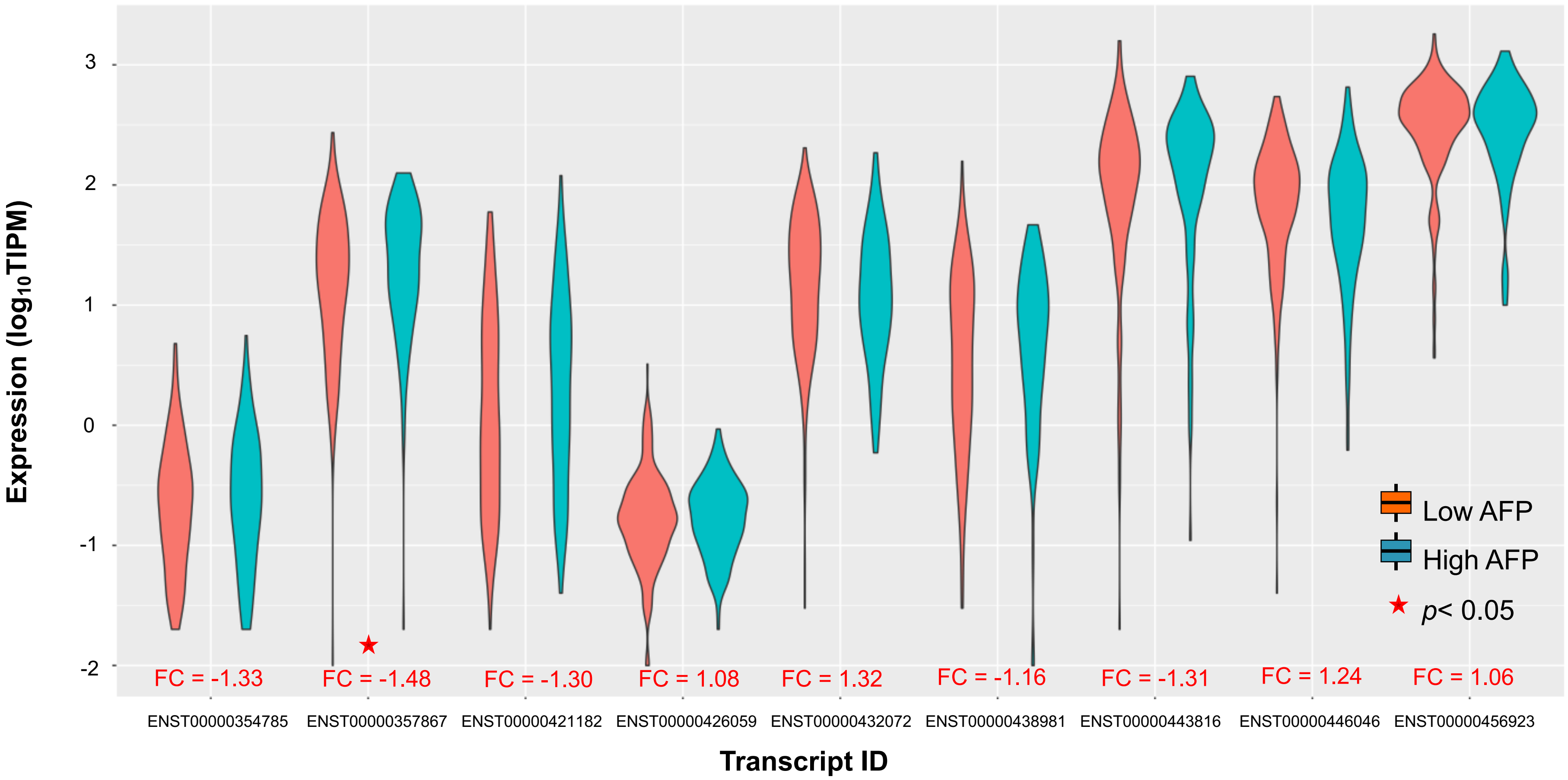

Figure S4. Expression of *FN1* mRNA transcripts between low and high AFP groups. We selected prot ein coding transcripts for which the median value of expression in total samples was more than zero and c ompared expression between low (<20 ng/mL) and high (>=20 ng/mL) serum AFP groups with a *t*-test. Fol d changes were calculated as the ratio of mean expression and tested by *t*-test.

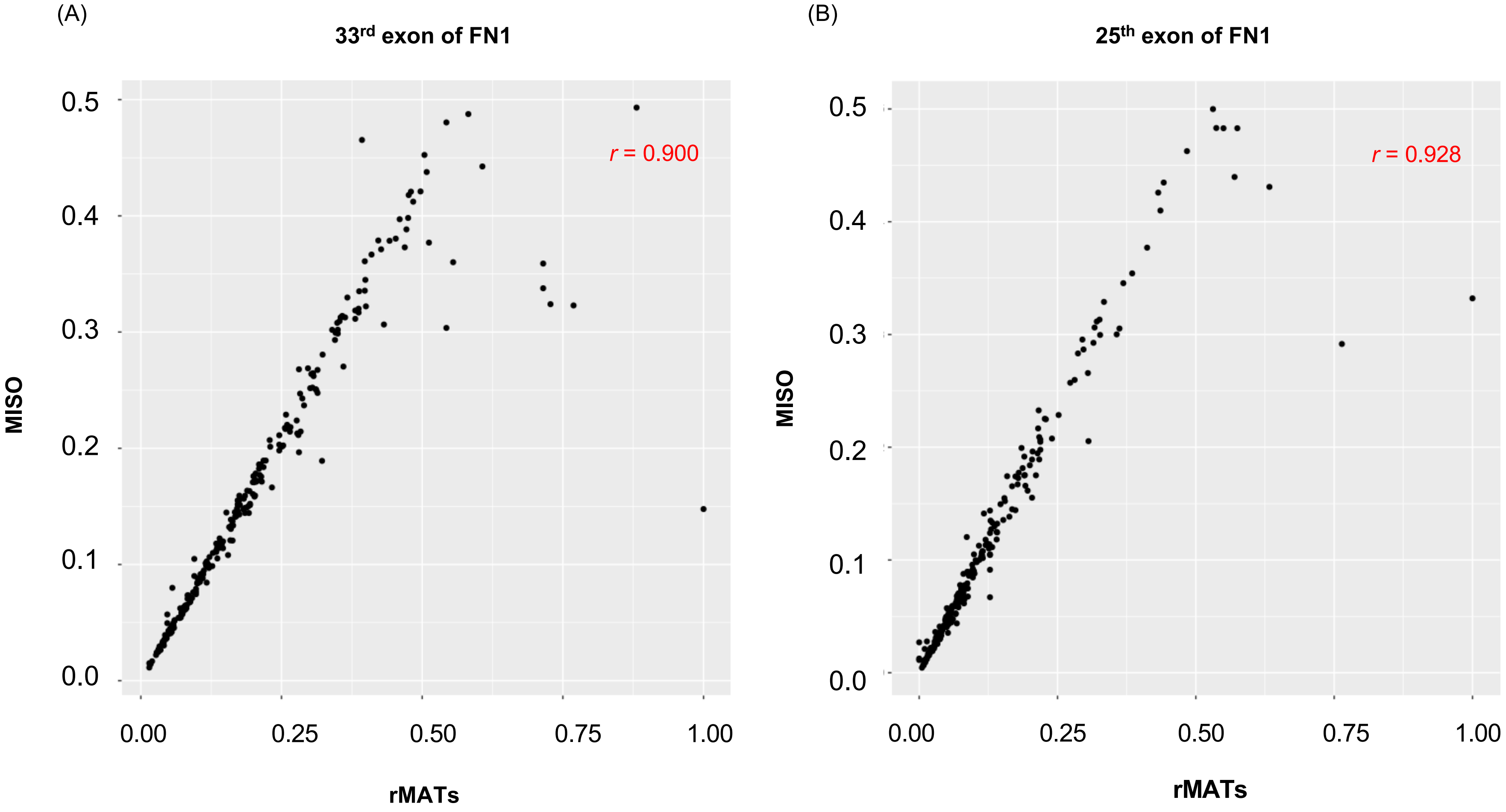

Figure S5. High correlation of PSI values between two different AS quantification tools. (**A**) A scatter plot of PSI level of 33rd exon in FN1 calculated from x-axis (rMATs) and y-axis (MISO). The correlation is 0.900. (**B**) A scatter plot of PSI level of 25th exon in FN1 calculated from x-axis (rMATs) and y-axis (MISO). The correlation is 0.928.
